# *Chlamydia trachomatis *responds to heat shock, penicillin induced persistence, and IFN-gamma persistence by altering levels of the extracytoplasmic stress response protease HtrA

**DOI:** 10.1186/1471-2180-8-190

**Published:** 2008-11-06

**Authors:** Wilhelmina M Huston, Christina Theodoropoulos, Sarah A Mathews, Peter Timms

**Affiliations:** 1 Institute of Health and Biomedical Innovation, 60 Musk Ave, Queensland University of Technology, Kelvin Grove, QLD, 4059, Australia; 2 Analytical and Electron Microscopy Facility, Queensland University of Technology, Brisbane, QLD, 4001, Australia

## Abstract

**Background:**

*Chlamydia trachomatis*, an obligate intracellular human pathogen, is the most prevalent bacterial sexually transmitted infection worldwide and a leading cause of preventable blindness. HtrA is a virulence and stress response periplasmic serine protease and molecular chaperone found in many bacteria. Recombinant purified *C. trachomatis *HtrA has been previously shown to have both activities. This investigation examined the physiological role of *Chlamydia trachomatis *HtrA.

**Results:**

The *Chlamydia trachomatis htrA *gene complemented the lethal high temperature phenotype of *Escherichia coli htrA*^- ^(>42°C). HtrA levels were detected to increase by western blot and immunofluorescence during *Chlamydia *heat shock experiments. Confocal laser scanning microscopy revealed a likely periplasmic localisation of HtrA. During penicillin induced persistence of *Chlamydia trachomatis*, HtrA levels (as a ratio of LPS) were initially less than control acute cultures (20 h post infection) but increased to more than acute cultures at 44 h post infection. This was unlike IFN-γ persistence where lower levels of HtrA were observed, suggesting *Chlamydia trachomatis *IFN-γ persistence does not involve a broad stress response.

**Conclusion:**

The heterologous heat shock protection for *Escherichia coli*, and increased HtrA during cell wall disruption via penicillin and heat shock, indicates an important role for HtrA during high protein stress conditions for *Chlamydia trachomatis*.

## Background

HtrA is a highly conserved serine protease and chaperone found in both eukaryote and prokaryote organisms (reviewed [1]. *Escherichia *(*E.) coli htrA *was identified as essential for growth at temperatures higher than 42°C (high temperature requirement) [2], and as the locus required for degradation of misfolded proteins (hence it is also referred to as DegP) [3]. HtrA has since been reported to be a periplasmic protease and chaperone during *E. coli *extracytoplasmic stress response, with a structural temperature switch to mediate between these two activities [4-6]. HtrA has important functions for virulence and stress resistance in a variety of bacteria (reviewed [7]). Recently we characterised purified recombinant HtrA from *Chlamydia *(*C*.) *trachomatis *L2, demonstrating that it had biochemical features typical of a HtrA protease, and critically, that it was capable of both protease and chaperone activities at physiologically relevant temperatures [8]. The protease activity was temperature activated (≥ 34°C) and specific for unfolded proteins. However, the physiological function of HtrA during the developmental cycle of *Chlamydia *is currently unknown.

*Chlamydia *is an obligate intracellular bacterial pathogen, which is unable to be genetically manipulated, making traditional approaches such as gene deletion studies currently impossible. The bacterium undergoes a unique biphasic developmental cycle consisting of small (0.2 μm) extracellular, metabolically in-active, infectious particles called elementary bodies (EBs) and larger (0.8 μm–1.0 μm) intracellular, metabolically active particles termed reticulate bodies (RBs) (approximately 12–36 h post infection (PI) for *C. trachomatis *L2) (reviewed [9]). The RBs asynchronously reorganise back to EBs to enable continued infections. *htrA *expression may occur throughout much of the developmental cycle of *C. trachomatis*, as transcripts for the gene were detected from 8 h to 40 h PI, with much higher expression levels occurring later in development [10].

*Chlamydia *can also enter a persistent phase of development whereby the RBs morphologically and metabolically adapt to remain indefinitely within the host cells, often in response to nutrient deprivation or other stress conditions [11-13]. Persistence induced by the presence of the cytokine IFN-γ, thought to correlate with *in vivo *chronic infections, has been widely used in laboratory studies to investigate the molecular basis of persistence [13]. Depletion of host cell pools of tryptophan via IFN-γ induction of indoleamine-2,3-dioxygenase, the first enzyme in the catabolism of this amino acid has been characterised as the mechanism leading to persistence of *C. trachomatis *[14]. The IFN-γ persistent *Chlamydia *typically are larger morphologically altered RBs, with fewer cells present in each inclusion and do not produce EBs [13]. The effect of IFN-γ treatment on *C. trachomatis *transcriptome was analysed by Belland and coworkers [15], who reported that HtrA transcript levels during IFN-γ persistence were approximately 2 fold less than acute conditions at 24 h PI and were present at similar levels to acute cultures at 48 h PI. The gene for the major outer membrane protein (*ompA *gene, MOMP) transcript was also reduced during IFN-γ persistence [15]. MOMP and LPS proteins were reported to decrease during IFN-γ persistent cultures of *C. trachomatis *although the decrease was most noticeable at time points beyond the scope of this investigation [13,14]. In contrast, HtrA protein levels increased during IFN-γ persistent infection models with *C. pneumoniae *[16]. Furthermore, a key role of HtrA during heat stress was also suggested by the detection of 9.6 fold increased levels of HtrA during a proteomic analysis of heat shock response in *C. pneumoniae *[16]. Penicillin is also known to induce persistence of *C. trachomatis *by inhibition of binary fission of RBs from approximately 12 hours into development, although chromosome and plasmid replication continues, and enlarged RBs are formed in large inclusions [17-19].

In the absence of a transformation system, genomic and proteomic approaches have been used to probe the function of many genes throughout the developmental cycle and persistence cultures of *Chlamydia*. In the present study, we have analysed *C. trachomatis *L2 HtrA during development, persistence, and heat shock conditions using polyclonal sera generated against purified recombinant HtrA to test the protein levels by western blotting and immunocytochemistry. The ability of the *C. trachomatis *L2 *htrA *to complement the heat sensitive phenotype of *E. coli htrA*^- ^was also demonstrated.

## Results

### HtrA is highly conserved between *Chlamydia *species and is homologous to *E. coli *HtrA

**Figure 1:**
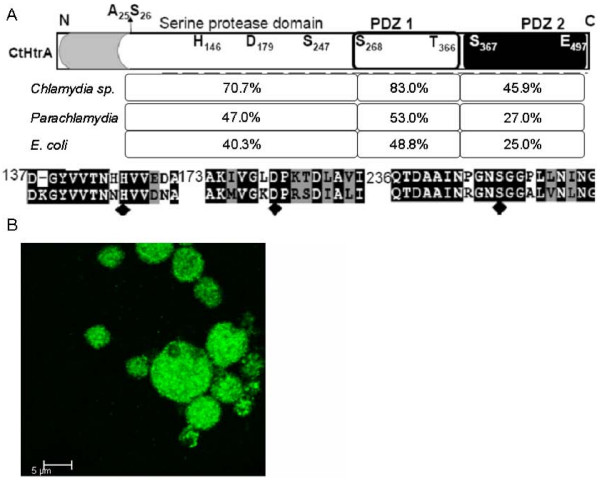
**HtrA conservation between *Chlamydia *and *E. coli *shows likely functional redundancy between bacterial species.** (A) Schematic of the HtrA protein from *C. trachomatis *is shown. The predicted N-terminal secretion signal sequence (shaded) and signal protease cleavage site (A25-S26), serine protease domain and two C-terminal PDZ domains are shown. The percentage of conservation of amino acid sequences (identical residues) for each of these regions between *Chlamydia *species, between all *Chlamydia *and *Parachlamydia*, and between *E. coli *HtrA and *C. trachomatis *HtrA (*E. coli*) are indicated below. An alignment of the residues surrounding the three essential residues for serine protease catalytic triad (H146, D179, S247; diamonds) from *C. trachomatis *L2 and *E. coli *HtrA is shown. (B) Immunohistochemistry using HtrA polyclonal sera with HEp-2 cells infected with *C. pneumoniae *AR39 at 72 h PI. *C. pneumoniae *AR39 infections were centrifuged for 30 mins at 2000 rpm and media changed at 2 h PI to media with 1 mg/ml cyclohexamide. Cells were fixed and stained as described in Methods.

There is considerable knowledge of the key residues and domains involved in the protease and chaperone activities for HtrA from *E. coli *(EcHtrA), and high conservation of these residues in other bacterial HtrA homologs has been reported [20-22]. HtrA is highly conserved throughout the *Chlamydia *species (sp.) as multiple sequence alignment demonstrated 64% identity and 17% similarity of the amino acid sequences (Fig. 1). The high homology of HtrA between the *Chlamydia *sp. is supported by the ability of the polyclonal sera generated to recombinant *C. trachomatis *L2 HtrA (this study, CtHtrA) to detect *C. pneumoniae *AR39 HtrA by immunofluorescence (Fig. 1B). The three essential residues for serine protease activity (Fig. 1A), and the two putative C-terminal PDZ domains are conserved in all of the *Chlamydia *HtrA homologs from the available genomes. PDZ domains function in protein-protein interaction and protein binding in a range of different proteins [23]. PDZ1 sequence is highly conserved between chlamydial HtrAs, functions in *E. coli *HtrA for coordinating substrate binding and access to the active site, including by binding of the carboxyl terminal of the protein substrates, PDZ2 is known to function in protein interaction within the *E. coli *HtrA hexameric structure [23,24]. The homology between the ancestoral relative *Parachlamydiaceae *HtrA sequences and *Chlamydia *was found to be not a lot greater than the homology to *E. coli *HtrA, suggesting whilst key features remain conserved, HtrA has evolved along with the *Chlamydia *genomes to suit the intracellular niche. The predicted cellular location of HtrA is within the periplasm for all *Chlamydia sp*, as determined using PSORT andSignalP [25,26]. This suggests that *Chlamydia *HtrA has a similar physiological function to the homologs in other bacteria for maintenance of extracytoplasmic protein quality, by both protease and chaperone activities.

### *C. trachomatis htrA *can complement the lethal high temperature phenotype of *E. coli htrA*^-^

The increased levels of HtrA during *C. pneumoniae *heat shock [16], and the essential role for *E. coli htrA *during growth at temperatures higher than 42°C [2], have led us to test the ability of *Chlamydia *HtrA to complement the heat sensitive phenotype of *htrA*^- ^*E. coli*. A *htrA*^- ^mutant was generated in *E. coli *MG1655 for the purposes of this investigation using the λ red recombinase method as described by Datensko and Wanner [27]. The *htrA*^- ^strain was verified by PCR analysis using primers to the Kan cassette and to the chromosome flanking the *htrA *gene (primer sequences shown in Methods, data not shown), the strain showed the expected phenotype of lack of growth at 44°C when tested on LB agar plates, whereas the wild-type (MG1655) was able to grow (data not shown). The ability of the *E. coli htrA *(*echtrA*) and *C. trachomatis htrA *(*cthtrA*) genes supplied on the low copy number vector pACYC184 (constructed as described in Methods) to complement the heat sensitive phenotype of the *E. coli htrA*^- ^mutant was tested. When *E. coli htrA*^- ^was complemented with either *E. coli *or *C. trachomatis htrA *(*htrA*^- ^pACYCechtrA and *htrA*^- ^pACYCcthtrA respectively) growth was restored at 44°C. The *E. coli htrA*^- ^strain harbouring only pACYC184 (*htrA*^- ^pACYC184) was unable to grow at 44°C on LB agar plates (data not shown). Thus, both the *E. coli htrA *and *C. trachomatis htrA *genes are able to complement the heat stress phenotype of *E. coli htrA*^-^.

**Figure 2:**
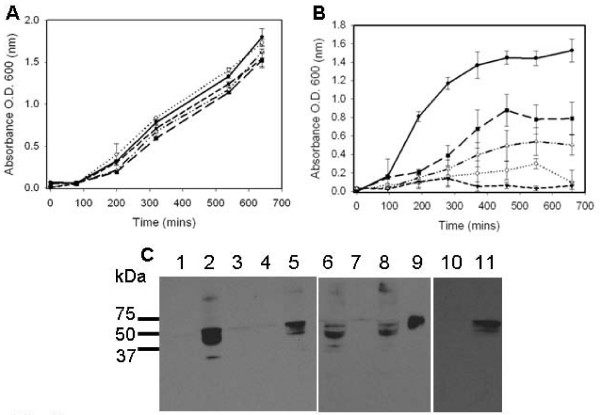
**Growth curve analysis of the complementation of *E. coli htrA*^- ^temperature sensitive phenotype by *C. trachomatis htrA*.** (A. B.) Growth of *E. coli *strains at 30°C (A) and 44°C (B). Samples are: Solid line; MG1655: wild-type *E. coli*,; dashed line: *htrA*-, dotted line: *htrA*-pACYC184, dashed and dotted line; *htrA*-pACYCEcHtrA, large dashed line, *htrA*-pACYCCTHtrA. Triplicate cultures were conducted for each experiment, with standard deviations indicated by the error bars. (C) Western blot of the total *E. coli *extracts for CtHtrA protein. Samples are: (1–8: 30°C) 1: *E. coli *MG1655, 2: *E. coli *MG1655 pACYCcthtrA, 3: *E. coli htrA*-pACYC184, 4: *E. coli htrA*-pACYCechtrA, 5: *E. coli htrA*-pACYCcthtrA, (6–9 44°C) 6: *E. coli *MG1655 pACYCcthtrA, 7: *E. coli htrA*-pACYCechtrA, 8: *E. coli htrA*-pACYCcthtrA, 9: purified recombinant HtrA protein. *Chlamydia *culture samples are included; 10. total extract HEp-2 cells, 11. total extract HEp-2 *C. trachomatis *L2 infected (20 h PI).

During the initial stages of this investigation the *C. trachomatis htrA *was supplied on the higher copy number vector pBR322 which proved toxic to *E. coli *at 30°C, and was unable to complement the heat sensitive phenotype (data not shown). This finding is consistent with previous reports of a similar copy number dependent toxicity of the *E. coli htrA *gene during complementation studies [2]. In order to ensure that no toxicity occurred with either *C. trachomatis *or *E. coli htrA *genes, the strains were further tested by determining growth curves at 30°C and 44°C on LB broth. The presence of either pACYCechtrA or pACYCcthtrA did not alter the growth of *htrA*^- ^*E. coli *at 30°C aerobically on LB media (Fig. 2A), similarly no effect of the complementation plasmids was observed for the growth of wild-type MG1655 (data not shown). The complementation plasmids were not able to completely restore the growth of *htrA*^- ^to that of wild-type when grown aerobically at 44°C (LB media) (Fig. 2B), however the strains were able to grow under these conditions where *htrA*^- ^is lethal, demonstrating that on both liquid media and agar plates the *C. trachomatis htrA*^- ^gene was able to complement the lethal high temperature phenotype of *E. coli htrA*^-^.

In order to confirm that the complementation was due to the presence of *C. trachomatis *HtrA protein (CtHtrA), western blots were conducted on the total soluble extracts from the *E. coli *strains using the polyclonal sera generated for this investigation. The CtHtrA protein was present in the MG1655 pAYCYcthtrA (lanes 2 and 6) and *htrA*^- ^pAYCYcthtrA (lanes 5 and 8) strains grown at both 30°C and 44°C (Fig. 2C). The CtHtrA, protein which is predicted to be 50.6 kDa, appears as a doublet on the western blot at approximately 50 kDa, which is likely due to the mature and immature forms of the protein where the signal sequence is not yet cleaved. The Figure also shows a western blot with *C. trachomatis *uninfected and infected HEp-2 cell extracts to demonstrate the specificity of the sera to a band at the expected size for CtHtrA (Fig. 2C, lanes 10 and 11).

### HtrA is present at higher levels in *Chlamydia trachomatis *L2 during heat stress

**Figure 3:**
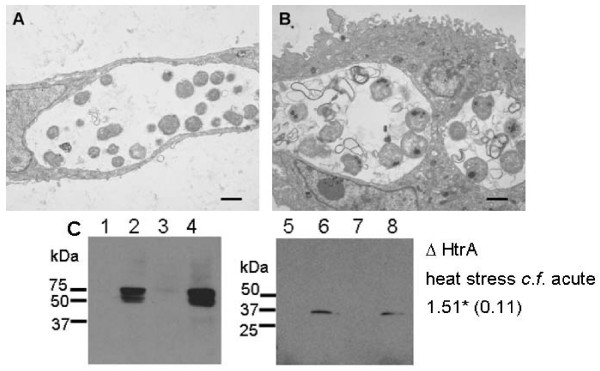
***C. trachomatis*****L2 response to heat shock includes increased levels of the HtrA protein and significant morphological alterations.** (A) Transmission electron micrographs (TEM) of a *C. trachomatis *L2 infected HEp-2 cell (acute infection) at 23 h PI. (B) TEM of a *C. trachomatis *L2 infected HEp-2 cell which was shifted to 42°C for 3 h at 20 h PI (23 h PI). (C) Western blots for HtrA and MOMP against total cellular extracts from the acute and heat shock cultures. Samples are (HtrA polyclonal sera) 1: uninfected HEp-2 cells acute conditions, 2: *C. trachomatis *L2 infected HEp-2 cells acute (23 h PI), 3: uninfected HEp-2 cells heat shocked for 3 h at 42°C at 20 hours post infection, 4: *C. trachomatis *L2 infected HEp-2 cells heat shocked for 3 h at 42°C at 20 h PI; (MOMP monoclonal antibody) 5: uninfected HEp-2 cells acute conditions, 6: *C. trachomatis *L2 infected HEp-2 cells acute (23 h PI), 7: uninfected HEp-2 cells heat shocked for 3 h at 42°C at 20 h PI, 8: *C. trachomatis *L2 infected HEp-2 cells heat shocked for 3 h at 42°C at 20 h PI. The quantification of HtrA western blot band intensity was conducted using densiotometry, three separate experiments examined by western blot were used to quantify the difference in band intensities between acute and heat shock samples. The average of these three differences (heat shock:acute) is indicated to the right of the figure with the standard deviation indicated in parentheses.

The increased levels of the HtrA from *C. pneumoniae *heat stress cultures [16], and the ability of *C. trachomatis *HtrA to complement the heat sensitive phenotype of *E. coli htrA*^- ^both suggest a critical role for HtrA in the *Chlamydia *heat stress response. The expression of HtrA from *C. trachomatis *during heat stress was tested using using *C. trachomatis *L2 infected HEp-2 monolayers at 20 h PI (post infection) which represents the mid-phase of *Chlamydia *development, when RBs are actively dividing. Cultures at 20 h PI were shifted to 42°C for 3 h and total cellular extract was immediately harvested; controls included uninfected HEp-2 cells and cultures which were not heat stressed. The cultures were examined by transmission electron microscopy (TEM) to determine the affect of heat stress on the morphology of the *Chlamydia *(Fig. 3A–B). The heat stressed cultures showed clear morphological alterations compared to untreated (acute) cultures, including: reduced numbers of RBs within the inclusion, larger RBs with condensation of electron opaque material within the RBs and excess membranous-like material within the inclusion.

**Figure 4:**
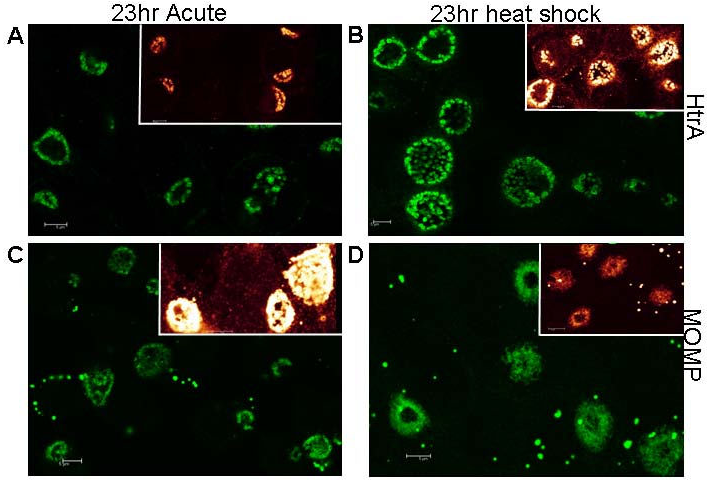
**Immunofluorescence intensity of HtrA is much higher in heat shocked *C. trachomatis*.** Immunocytochemistry on *C. trachomatis *L2 infected HEp-2 cells using antibodies to HtrA and MOMP. Immunofluorescence images collected using confocal microscopy with insert showing intensity differences (glow scale) from each experiment to demonstrate differences. Samples are (A) *C. trachomatis *L2 infected HEp-2 cells acute (23 h PI) (HtrA polyclonal sera). (B) *C. trachomatis *L2 infected HEp-2 cells heat shocked for 3 h at 42°C at 20 h PI (HtrA polyclonal sera) (23 h PI). (C) *C. trachomatis *L2 infected HEp-2 cells acute (23 h PI) (MOMP mAb). (D)*C. trachomatis *L2 infected HEp-2 cells heat shocked for 3 h at 42°C at 20 h PI (MOMP mAb). The inserts in the top right show relative intensities and were recorded under identical microscope and laser settings, on the same day, to enable comparison of levels of fluorescence. Scale bars (5 μM) are shown in the bottom left corner of each figure.

Total cellular extracts (loaded to equivalent protein amounts from total culture extracts) from the acute and heat stressed cultures were analysed by western blots for HtrA (Fig. 3C). Western blots for MOMP were conducted on the same samples to allow comparative analysis using densiotometry quanitification of band intensities in each sample (Fig. 3C). The localisation and expression of MOMP and HtrA was examined by indirect immunofluorescence microscopy (using the confocal laser scanning microscope, CLSM), where increased levels of HtrA immunofluorescence was detected during heat stress compared to acute conditions (Fig.4A–B). These results showed that MOMP protein levels decrease during heat stress (Fig. 4C), whereas the HtrA protein intensity showed a marked increase during heat stress when compared to acute conditions.

**Figure 5:**
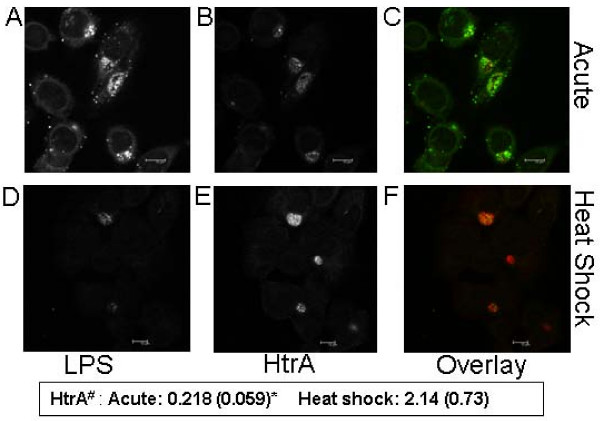
**Quantification of HtrA and LPS by dual labelled immunoflourescence during acute and heat shocked culture of *C. trachomatis *L2.** The Figure shows the immunoflourescence on co-labelled slides of LPS (A, D) HtrA (B, E) and overlays (C, F) during acute at 23 h PI (A-C) and heat shocked for 3 h at 42°C at 20 h PI (23 h PI). Quantification of the HtrA immunoflourescence (as a ratio of LPS immunoflouresence) under each condition is indicated in the box below the Figure. ^#^The quantification was conducted using the Leica software suite on individually selected inclusions with a minimum of 20 separate inclusions included in each analysis, *standard deviation is indicated in parantheses. The immunolabelling was conducted as per Methods, under identical laser conditions on the same day between the acute and heat shocked slides to allow quantification of fluorescence. Note: The argon laser to detect the emission of the FITC in (480–550 nm) collection region is at 100% laser power in these images due to the marked reduction in LPS present after heat shock, most other images presented in this paper with LPS-FITC labelling have been collected on 23% argon laser power. Scale bars (10 μM) are shown in the bottom right of each figure.

A similar trend was observed when HtrA immunofluorescence was compared to lipopolysaccharide (LPS) immunofluorescence during co-labelling experiments. LPS is significantly reduced during heat stress conditions (as shown in Fig. 5), whereas HtrA levels showed a marked increase (2.01 fold relative to LPS). These observations all support a key role for HtrA in the heat stress response by *C. trachomatis*.

### HtrA is likely to function during penicillin induced persistence of *C. trachomatis*

**Figure 6:**
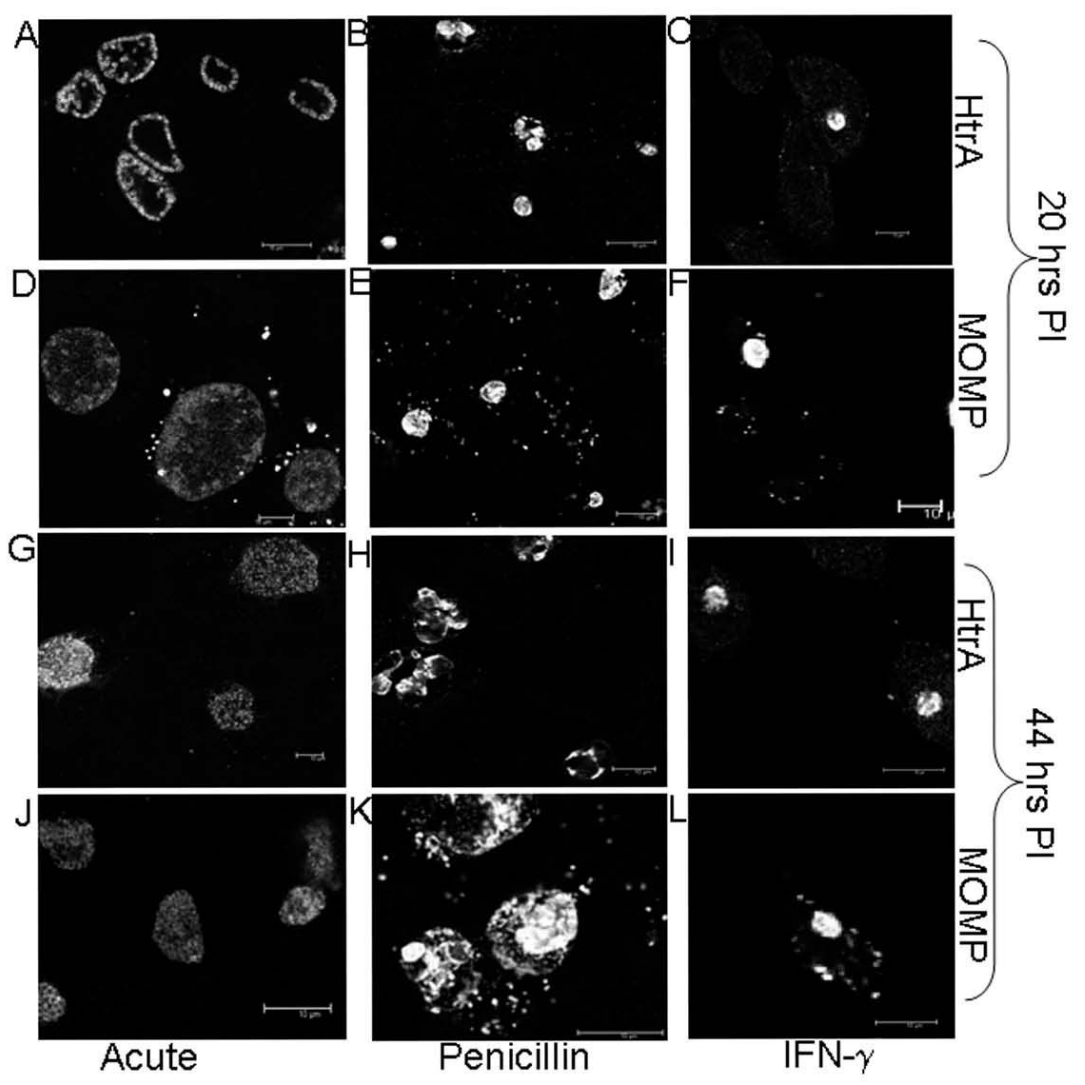
**Immunofluorescence of HtrA and MOMP during acute, penicillin and IFN-γ persistence culture models at 20 and 44 h PI. **The Figure depicts the confocal microscopy immunofluorescent images of HtrA (A-C, G-I) and MOMP (D-E, J-L) during different culture conditions of *Chlamydia trachomatis *L2, including; acute, penicillin persistence and IFN-γ persistence. Scale bars (10 μM) are shown in the bottom right of each figure.

Analysis of the HtrA cellular localisation under acute conditions using immunocytochemistry and confocal microscopy showed that the protein is located solely around the periphery of each reticulate body at 20 h PI; there was no immunofluorescence detected within the cytoplasmic space of the bacteria (Fig. 6A, Additional file 1). There are 6 inclusion vacuoles with numerous RBs within each inclusion visible in Fig 6A, each RB visible shows HtrA staining is only present around the periphery of the cell which supports extracytoplasmic location of HtrA, which could be periplasmic considering the *in silico *predictions. Similarly when closely examining every reticulate body within an inclusion vacuole HtrA staining was only visible around the periphery (Additional file 1; video of a series of Z sections). The labelling of the 44 h PI cultures with HtrA was consistent with the observations for 20 h PI with HtrA immunofluorescence also associated with the periphery of individual RBs. Additionally, increased intensity of HtrA immunofluorescence was associated with smaller particles corresponding to the size of both intermediate bodies (RB mid-conversion to EB) and EBs which would be expected to be present in the culture at this later stage of development (Fig. 6G). MOMP immunofluorescence was observed at 20 h PI and 44 h PI (Fig. 6D, J).

**Figure 7:**
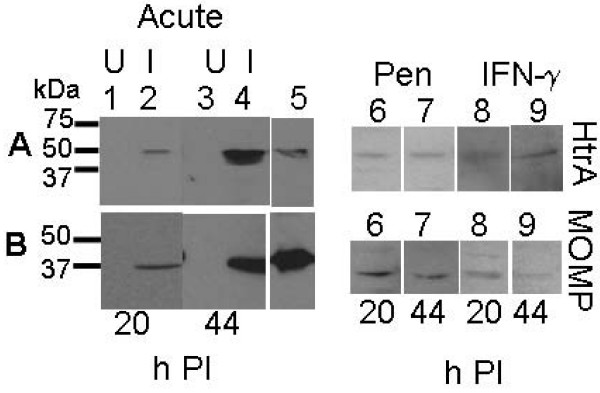
**Western blots for HtrA and MOMP proteins during acute, IFN-γ and persistence culture of *C. trachomatis *L2 to detect changes in protein levels.** (A) HtrA, (B) MOMP. Samples on the western blots are: 1. uninfected HEp-2 cell extract (20 h PI), 2. *C. trachomatis *infected HEp-2 cell extract (20 h PI), 3. uninfected HEp-2 cell extract (44 h PI), 4. *C. trachomatis *infected HEp-2 cell extract (44 h PI), 5. purified recombinant HtrA/MOMP (A/B respectively), 6. (20 h PI) Penicillin persistence model, 7. (44 h PI) Penicillin persistence model, 8. (20 h PI) IFN-γ persistence model, 9. (44 h PI) IFN-γ persistence model. Lanes 6–9 are shown in reduced size.

Immunocytochemistry and confocal microscopy of MOMP and HtrA revealed that both proteins were present during mid (20 h PI) and later (44 h PI) stages of penicillin persistence and resolved larger RBs contained within large inclusions (Fig. 6B, E, H, K) compared to the control acute cultures (Fig. 6A, D, G, J). This is consistent with previous reports of the *Chlamydia trachomatis *morphology during penicillin persistence [13,17]. HtrA and MOMP were also detected by western blot from the penicillin persistent cultures although less protein was detected than that observed for acute conditions (Fig. 7). The analysis of the persistent cultures by dual labelling immunocytochemistry for HtrA and LPS demonstrated that LPS is present during penicillin persistent cultures of *C. trachomatis *which is consistent with previous reports [28] (Fig. 9). Quantitative analysis of the fluorescence showed that HtrA was present at lower levels compared to acute cultures (as a ratio of LPS) at 20 h PI, but that higher levels of HtrA were present at 44 h PI (compared to acute cultures) (Table 1).

**Table 1:** Quantification of HtrA immunofluorescence (as a ratio of LPS) during penicillin and IFN-γ medicated persistence compared to untreated (acute) controls

**Acute**	**Penicillin**	**Acute**	**IFN-γ**
**20 h PI**		**20 h PI**	
1.1^a ^(0.08b)	0.84 (0.089)	0.74^&^(0.09)	0.62 (0.05)
**44 h PI**		**44 h PI**	
1.49 (0.23)	1.85 (0.052)	1.06 (0.04)	0.33 (0.13)

^a ^Each number represents the ratio of HtrA to LPS immunofluorescence in >10 individual inclusions dual labelled with LPS (FITC) and HtrA (561) antibodies. Quantification of total fluorescence was conducted under identical laser and microscopy settings on slides prepared on the same day (acute and persistence model).

^b ^Standard error measurements are shown in brackets beside each Figure.

^&^Immunofluorescence for IFN-γ cultures was conducted with a higher laser power setting for laser 561 collections to enable accurate calculations of the HtrA measurements. Due to this difference control acute ratios are shown for both conditions.

### HtrA levels are lower during IFN-γ persistence of *C. trachomatis *when compared to acute conditions

**Figure 8:**
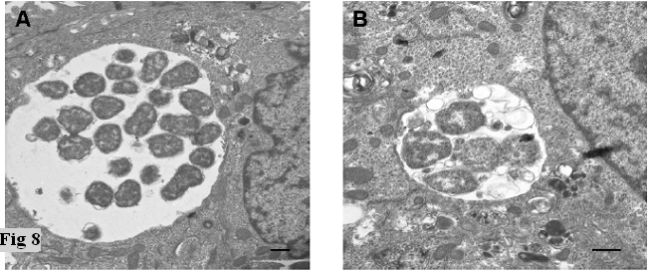
**Transmission electron micrographs of *C. trachomatis *infected HEp-2 cells.** (A) The figure shows a *C. trachomatis *infected inclusion under acute conditions at 20 hrs PI. (B) The figure shows a *C. trachomatis *infected inclusion within a HEp-2 cell in the presence of 50 U/ml IFN-γ at 20 hrs PI. TEM was conducted as described in Methods. The scale bar represents 1 μM.

**Figure 9:**
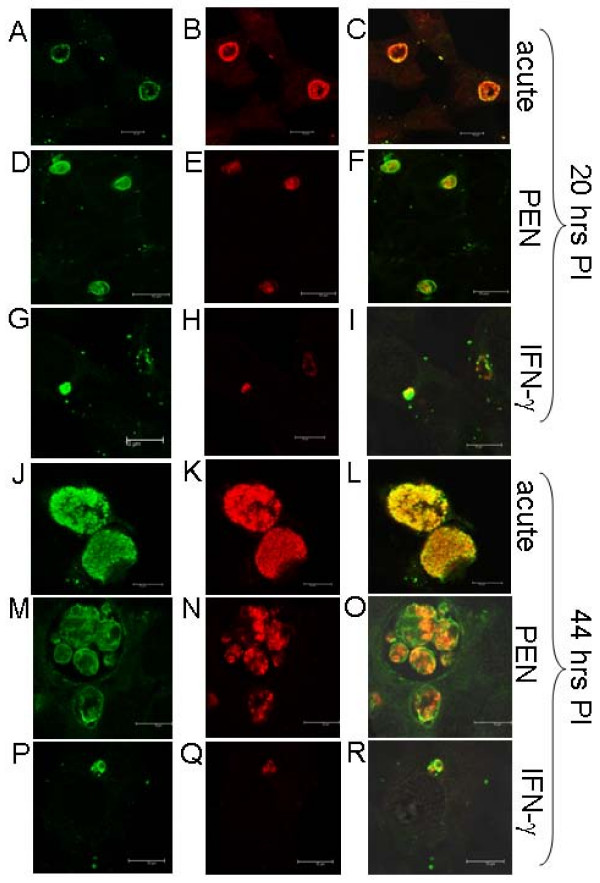
**Confocal microscopy images of dual labelled immunofluorescence of HtrA and LPS during *C. trachomatis *L2 HEp-2 cultures.** The figure shows immunocytochemistry of acute, penicillin persistent, and IFN-γ persistent cultures fixed and stained with HtrA and LPS antibodies at 20 h and 44 h post infection. The LPS antibody (shown in green, column 1) has a FITC label directly conjugated to the antibody, the HtrA antibody (shown in red, second column) was detected using a secondary antiRabbit IgG antibody with an Alexa Fluor label (561). Overlays are shown in the third column. Scale bars (10 μM) are shown in the bottom right of each figure.

HtrA, MOMP, and LPS were all detected by immunofluorescence at 20 and 44 h PI during IFN-γ persistence cultures during this investigation (Fig. 6C, F, I, L). The HtrA and MOMP proteins were also detected by western blot (Fig. 7). The MOMP and HtrA bands, which are less intense then that in the acute cultures, appear to decrease in intensities from 20 h PI to 44 h PI in the western blots, consistent with the change in transcript levels previously reported [15,18]. The acute and IFN-γ persistence cultures were examined by transmission electron microscopy (20 h PI) to determine if the RB morphology was comparable to that previously reported (Fig. 8), and the expected persistent morphology for RBs under these conditions was observed. HtrA immunofluorescence (as a ratio of LPS) from persistent cultures in the presence of IFN-γ showed markedly reduced levels compared to acute culture controls at both 20 h and 44 h PI (Fig. 9G, H, I, P, Q, R and Table 1).

## Discussion

HtrA is known to be an important stress response protease for many bacteria and has been shown to be critical for virulence in many bacteria, including intracellular pathogens *Salmonella enterica *and *Legionella pneumophila *[29,30]. There is considerable evidence from both microarray and proteomic studies that HtrA is expressed in *Chlamydia*.

In the absence of a genetic manipulation system, a complementation approach was used to test the physiological function of *C. trachomatis *HtrA in a heterologous host (*E. coli*). *E. coli *HtrA protein (EcHtrA) and *C. trachomatis *HtrA protein (CtHtrA) are known to have differences in substrate specificity for their protease activities, although both have temperature activated protease activity, and are specific for unfolded proteins [4,8]. The findings reported here show that the *C*. *trachomatis htrA *was able to protect *E. coli htrA*^- ^against its lethal high temperature phenotype. This suggests that the ability to chaperone and degrade unfolded proteins, regardless of specificity for residues at the site of peptide bond cleavage, is sufficient to protect against the damage caused by heat stress. Minor differences observed in complementation by *echtrA *and *cthtrA *(Fig. 2) could be attributed to; differences in substrate specificities of the enzymes, potential differences in the 'switch' to chaperone activity at higher temperatures as *E. coli *has been previously reported to act only as a chaperone at temperatures below 30°C (Speiss et al., 1999) although more recent studies suggest EcHtrA may have chaperone activity at higher temperatures (Skorko-Glonek *et al*., 2007), or finally due to different copy number requirements for the two genes. However, regardless of the minor differences in complementation by growth curve, it is clear that *Chlamydia htrA *can protect *E. coli htrA*^- ^against the detrimental affects of heat stress. This data provides direct evidence for *in vivo *physiological functionality of *C. trachomatis *HtrA as a molecular chaperone and/or protease to protect against protein stress induced by high temperatures. The lack of a genetic manipulation system for *Chlamydia *limits the ability to collect *in vivo *physiological evidence, however we feel the use of this heterologous system has provided strong evidence that *Chlamydia *HtrA protects against protein stress *in vivo*.

Protein levels were examined in *C. trachomatis *L2 cultures under acute and heat stressed conditions to examine the role of the HtrA. Heat stress is an ideal laboratory model to induce protein level stress and is highly relevant for many bacteria, but it is important to note that *C. trachomatis *would more commonly encounter other forms of protein damaging stress such as, immune related oxidative and nitrosative stress, temperature flux in the genital tract, rapid pH changes during early development of the inclusion vacuole, and possibly osmotic flux. The increased level of HtrA during heat stress was observed both by western blot and immunofluorescence (relative to MOMP and LPS). The use of the major outer membrane protein (MOMP) as a comparative protein for molecular studies in *Chlamydia *is widely reported. MOMP levels have been reported to decrease during *C. trachomatis *persistence and stress conditions [15,18]. However, as different fixatives were required for the HtrA and MOMP antibodies used during this study, LPS was used in conjunction with HtrA immunofluorescence. The heat stress model we tested was consistent with previous studies on *C. pneumoniae *and *C. trachomatis *with both morphological similarity when examined by TEM and similar decreased levels of MOMP and LPS [14,16,17]. Furthermore, we tested under conditions for which it is known a high proportion of the *C. trachomatis *cells remain viable. That is, after 3 hrs heat shock at 42°C once heat stress is removed, *C. trachomatis *was able to complete the developmental cycle, and form infectious EBs [31]. This suggests that CtHtrA is important during heat stress and could be one of the key factors for protection of cellular envelope proteins sufficiently to allow restoration of normal growth once heat stress is removed. The protection is likely to be mediated by both the protease and chaperone functions we previously reported during *in vitro *investigations of HtrA [8]. The data presented here clearly indicates a cell envelope or extracytoplasmic localisation of HtrA which seems likely to be periplasmic as indicated by in silico predictions. Further experimental data would be needed to confirm the exact location of HtrA (periplasmic, cytoplasmic membrane, outer membrane), however, regardless of the exact location it is clear that HtrA's protein maintenance function would still serve much of the extracytoplasmic proteome.

Two well established persistence models were used during this investigation to test for the potential significance of HtrA for persistent infections. The penicillin induced persistence model affects the development of *Chlamydia *by binding to three penicillin binding proteins halting binary fission and preventing later phases of the chlamydial developmental cycle (formation of EBs)[17,32]. This affect of penicillin on *Chlamydia *is somewhat paradoxical as there is no unequivocal evidence for the presence of peptidoglycan, although it is predicted that peptidoglycan synthesis functions in RBs during chlamydial cell division (reviewed [33]). Thus, in the presence of penicillin at 20 h PI, the RBs will have no peptidoglycan (PG) or components thereof, and less MOMP and outer membrane cysteine rich proteins such as OmcB [18,19] which normally contribute to the rigidity of the outer membrane by extensive disulfide crosslinking. The decreased outer membrane disulfide crosslinking and peptidoglycan likely results in reduced extracytoplasmic integrity, reduced protection from osmotic and redox stress for extracytoplasmic proteins due to these reduced physical and chemical barriers. Osmotic and redox stress is known to affect protein integrity [34]. Thus, HtrA may be present at increased levels during penicillin persistence to protect against this possible additional protein stress. These findings suggest that HtrA is less important for the initial adaptation to penicillin but rather functions during longer term persistence presumably via maintenance of extracytoplasmic proteins possibly required for ongoing viability or for the capability to return to normal development once the selective pressure is removed.

HtrA protein levels were reduced at both 20 h PI and 44 h PI during IFN-γ persistence, possibly indicating that extracytoplasmic protein stress is less important during this form of persistence until at least 44 h PI. IFN-γ persistence for *Chlamydia *is effectively a form of amino acid deprivation stress, which results in a lack of binary fission and prevention of RB development to EBs. The amino acid deprivation is not likely to result in direct stress on existing extracytoplasmic proteins, which could explain the reduced levels of HtrA under these conditions. The considerable reduction in HtrA during IFN-γ persistence, when compared to acute, heat stress and penicillin data, suggests that IFN-γ persistence doesn't involve chlamydial extracytoplasmic stress response, unlike previous suggestions for *C. pneumoniae *that IFN-γ persistence is a stress response [16].

## Conclusion

The role of HtrA for *Chlamydia *likely involves both the protease and/or chaperone functions in protection, assembly, or degradation, of extracytoplasmic chlamydial proteins during intracellular development (RB), extracellular survival (EB), and stress conditions. The data presented here demonstrated the presence of HtrA during development, with increased HtrA levels during conditions which most likely induce protein stress, such as heat shock and penicillin induced persistence. Furthermore, the results suggest that IFN-γ persistence doesn't involve an extracytoplasmic stress response for *Chlamydia trachomatis*. These observations combined with the demonstrated ability of *Chlamydia *HtrA to protect *E. coli htrA*^- ^against lethal heat stress *in vivo*, support an important role of HtrA for maintaining the viability of *Chlamydia *during any conditions where extracytoplasmic protein stability is compromised. The findings of this study have further contributed to the understanding of the role of HtrA for bacterial pathogenesis [29,35], particularly within the intracellular niche.

## Methods

### Bacterial culture and media

*E. coli *were cultured using LB media [36] aerobically at 37°C, aerobic broth cultures were shaken at 220 rpm. All cloning experiments and plasmid construction were conducted and maintained in JM109. Genetic mutation and physiological experiments were conducted using *E. coli *MG1655. All chemicals were sourced from Sigma-Aldrich unless otherwise stated.

### Cell culture and *Chlamydia *culture

The human epithelial cell line (HEp-2) were cultured in DMEM media supplemented with 5% fetal calf serum, 50 μg/ml gentamycin, and 10 μg/ml streptomycin, at 37°C 5% CO_2_. All *C. trachomatis *L2 infections were established at 70–90% infectivity and media was changed at 4 h post infection to media containing 1 μg/ml cyclohexamide. Heat stress experiments were performed in an incubator at 42°C, 5% CO_2_. Penicillin persistence was established by addition of penicillin at time of media change (150 mins PI) to a final concentration of 50 U/ml. IFN-γ was added to the HEp-2 cells 24 h prior to infections at 50 U/ml and maintained throughout the experiment with media changes every 24 h.

### Genetic manipulations, PCRs and plasmid constructs

*E. coli *MG1655 *htrA*-mutant was generated using the λ red recombinase method essentially by the method described by Datensko and Wanner (2000). The pDK13 template plasmid (kanamycin resistance) was used with the primers EchtraF4 5'-ttgtaaagacgaacaataaatttttaccttttgcagaaactttagttcgtcaaacatgagaattaattccgggg-3' and EchtraR4 5'-aagatgccagccagccataagtcctccgttatgcacggcttagcataaggcatatgaatatcctccttag-3. The PCR product was used to electroporate MG1655 *E. coli *containing the λ red recombinase expression plasmid pKD46. Expression of the recombinase was induced with 10 mM arabinose during culture of the cells for electrocompetency preparation. The *E. coli htrA *gene was mutated by insertion/deletion of the *htrA *open reading frame with the PCR product, regions of homology with the *E. coli *genome for the sites of recombination are shown underlined in the above primers. The mutation was confirmed using PCR analysis of purified genomic DNA using the primers K1 5'-tgcagttcattcagggcaccg-3', K2 5'-atgcccgacggcgaggatc-3', EcHtrAf5 5'-tgaccgacctctatgcgtgg-3' and EcHtraR5 5'-atggtacgtcggacgatatcc-3' to show insertion of the kanamycin resistance cassette into the genome in the place of the *htrA *gene.

The complementation plasmids were constructed using PCR products generated from purified genomic DNA isolated from *E. coli *MG1655 or *C. trachomatis *L2. pACYCechtrA was generated by PCR of the *htrA *gene from *E. coli *using the primers echtrAf6 5'-gcggatccatggccgtagaacaataacccagg-3' and echtraR6 5'-ccgcatgcataaggaagtacgtaacgtaccgg-3'. The *E. coli htrA *PCR product was cloned into the pACYC184 vector by double digest using the primer incorporated restriction endonuclease sites for BamH1 and Sph1 (underlined). A similar strategy was employed to generate the pACYCcthtrA complementation vector, whereby the PCR product was amplified using the primers cthtraF10 5'-ggggatccttggagaatcatcagagtag-3' and cthtraR2 5'-ttgcatgcctactcgtctgatttcaagacgatg-3'. The PCR product was cloned into pACYC184 using the restriction endonucleases BamH1 and Sph1, primer incorporated sites (underlined). All PCR reactions were conducted using Pfu DNA polymerase (Promega, Australia) as per the manufacturer's instructions. Plasmid constructs were confirmed by restriction enzyme digest and sequence analysis on both strands prior to transformation into *E. coli *MG1655 and *E. coli htrA*^- ^using electroporation.

### Bioinformatic analysis of protein sequences

Bioinformatic analysis was conducted using the following programs; EclustlW [37], key residues were identified by alignment with *E. coli *HtrA using MULTALIN [38], PDZ domains predicted by MOTIFSCAN [39], prediction of subcellular localisation was conducted using the program PSORT [25].

### Generation of polyclonal sera

Polyclonal sera against recombinant purified HtrA [8] was commercially generated by IMVS (South Australia). Rabbits were injected with 200 μg of purified HtrA protein in Freunds complete adjuvant, with a further three boosts using 200 μg purified HtrA protein in Freunds incomplete adjuvant.

### Western blots and preparation of protein extracts

*E. coli *total extracts were prepared by harvesting cells by centrifugation at 4000 × g for 10 mins. Cells were washed and resuspended in PBS (0.1 volume), and lysed by sonication (Misonix 3000) and total protein concentration determined using the BCA assay (Sigma-Aldrich, Australia). Total extract from HEp-2 cell cultures (infected and uninfected) were harvested from monolayers conducted in tissue culture flasks. Media was removed and cells washed in cold PBS. Cells were scraped into a small volume of PBS (0.05 original media volume, identical volumes used for acute and test conditions) and lysed by sonication prior to quantification of total protein extract. Polyacrylamide gel electrophoresis (PAGE) was conducted to test protein harvests and for western blots. Samples were loaded to identical protein concentrations from total protein extracts for western blot analysis. Western transfers were conducted using Hybond-C (GE Healthcare, Australia). Western blots to analyse *E. coli *extracts were blocked in 0.5% skim milk powder in TBS, HtrA polyclonal sera was used as primary antibody at 1/7000 dilution (0.5% skim milk powder, TBS). Anti-rabbit HRP conjugate antibody (Sigma-Aldrich) was used as the secondary antibody (1/10000) and ECL Plus Western Blotting Detection System (GE Healthcare, Australia) was used for detection. Western blot analysis of *Chlamydia *cell culture extracts were conducted using the same procedure, although 0.5% w/v casein (Roche, Australia) was used as the blocking agent and the HtrA polyclonal sera was used as primary antibody at 1/200 dilution and the MOMP monoclonal antibody was used at 1/1000. HRP conjugated secondary antibodies were used at 1/10000 dilution (Sigma-Aldrich, Australia). Recombinant MOMP was generated as described by Barker and coworkers [40].

### Transmission electron microscopy and immunofluorescence

Transmission electron microscopy was conducted on samples which were cultured concurrent to the experiments analysed by western blot. The cultures were fixed in 3% glutaraldehyde (ProSciTech), 0.1 M cacodylate buffer, pH 7.3. After overnight fixative cells were scraped and washed in 0.1 M cacodylate buffer. Cells were postfixed in 1% osmium tetroxide and embedded in Spurr expoy resin. Ultrathin sections (50 – 100 nm) were cut and stained with uranyl acetate and lead citrate stains prior to examination and photography using the JEOL 1200EX transmission electron microscope.

Immunocytochemistry samples were prepared using cells cultured on coverslips. Coverslips were washed in PBS prior to fixative for 15 mins (100% methanol for HtrA and LPS, 4% paraformaldehyde, PBS for MOMP). Cells were permeabilised by incubation in 0.5% v/v triton X-100, PBS for 15 mins prior to blocking in 1% w/v BSA for 20 mins. 0.1% BSA was used for LPS antibody staining and no permeabilisation step was used. Primary antibody was incubated for 1 hour in 1% BSA (LPS no dilution, HtrA 1/500 sera dilution, MOMP 1/1000 dilution). MOMP monoclonal antibody (MOMP mAb) was sourced from Biodesign International (C955135), LPS-FITC conjugated antibody was sourced from Cellabs, Australia. Washes were conducted using 0.2% Tween-20 PBS or PBS when the LPS antibody was used. Secondary antibodies were used at 1/600 dilution in 1% BSA for 1 hour. Secondary antibodies used were Alexa Fluor 488 goat anti-mouse and Alexa Fluor 488 goat anti-rabbit (Invitrogen, USA). Co-labelling with LPS and HtrA was conducted in 0.05% BSA, washed in PBS, and the HtrA antibody was detected using a secondary anti-rabbit IgG Alexa Fluor 568. Coverslips were mounted using Prolong Gold antifade (Invitrogen, USA) and viewed under a Leica TCS SP5 confocal laser scanning microscope (Leica Microsystems) with a 63 × oil objective 1.4 NA. Images were captured using the Leica application suite for advanced fluorescence. No immunofluorescence was detected in samples stained with either primary or secondary antibody alone. Quantification was conducted on the same day, to compare samples which were previously prepared (triplicate experiments; cultures, and slide preparations for direct comparisons were also conducted at the same time). Quantification of fluorescence was calculated using the Leica application suite.

## Authors' contributions

WMH conducted the experiments, microscopy experiments were conducted in conjunction with CT. WMH, SAM, and PT designed and conceived the study. WMH wrote the manuscript. All authors analysed results, read, and approved the manuscript.

## Supplementary Material

Additional file 1 **Video of series of Z-sections collected during immunocytochemistry of *C. trachomatis *L2 infected HEp-2 cell stained with HtrA polyclonal sera at 20 h PI (Alexa Fluor 488).** The video demonstrates the staining of HtrA was around the edge of each RB within this inclusion. The staining around the periphery of each RB was consistently observed at this time point indicating a likely periplasmic location of HtrA. Scale bar 10 μM. Click here for file
